# Pretreatment PSA levels affects the completion rate of Ra-223 treatment

**DOI:** 10.1038/s41598-021-86033-4

**Published:** 2021-03-19

**Authors:** Nobuko Utsumi, Hiromasa Kurosaki, Kosei Miura, Hiroki Kitoh, Koichiro Akakura

**Affiliations:** 1 Department of Radiation Therapy, Japan Community Healthcare Organization Tokyo Shinjuku Medical Center, 5-1, Tsukudocho, Shinjuku-Ku, Tokyo, 162-8543 Japan; 2 Department of Urology, Japan Community Healthcare Organization Tokyo Shinjuku Medical Center, 5-1, Tsukudocho, Shinjuku-Ku, Tokyo, 162-8543 Japan

**Keywords:** Prostate cancer, Radiotherapy

## Abstract

The aim of this study was to review our initial experience of using radium 223 (Ra-223) for metastatic castration-resistant prostate cancer (CRPC) and to evaluate whether pretreatment PSA levels correlate with completion of Ra-223 treatment. In addition, we examined change ratios of PSA, ALP and BAP after the third administration to evaluate the correlation of these change ratios with completion of the subsequent Ra-223 treatment. Forty patients were enrolled in this retrospective study. Ra-223 treatment was considered completed in patients who received five or six administrations. Patient backgrounds and changes in biomarkers were compared between patient groups (complete vs. incomplete Ra-223 treatment). PSA levels before treatment were significantly lower in the complete compared with the incomplete group (cutoff value; 21.7). ALP and BAP levels had decreased after the third administration in the complete group, compared with baseline levels, while levels in the incomplete group had increased. Significant difference was seen in ALP levels, while was not seen in BAP levels between the two groups. Ra-223 treatment should be considered for CRPC with low PSA levels. Changes in PSA and ALP during Ra-223 treatment might provide markers to identify patients likely to complete Ra-223 treatment, with implications for prognosis.

## Introduction

The incidence of prostate cancer in Japan has recently been increasing^1^. Prostate tumors initially depend on androgens for their growth and can therefore be controlled by surgical castration or by medical castration using luteinizing-hormone releasing agonists or antagonists (androgen-deprivation therapy), which is regarded as the standard of care for patients with advanced or metastatic disease in this setting. However, the disease progresses rapidly in many patients, commonly referred to as castration-resistant prostate cancer (CRPC)^2–4^. Most patients with CRPC (90%) have radiological evidence of bone metastasis, which is the primary cause of disability, reduced quality-of-life, and death. Bone metastases cause pain and skeletal-related events, including pathological fractures, spinal cord compression, and bone marrow deficiency.

Novel therapies have been approved for the treatment of metastatic CRPC (mCRPC) over the past decade, including the bone-targeting agent radium-223 dichloride (Ra-223). In May 2013, the US Food and Drug Administration approved the use of this radiopharmaceutical for the treatment of CRPC with symptomatic bone metastases and unknown visceral metastatic disease, and Ra-223 was subsequently approved in Japan in March 2016, since when it has become available for the treatment of mCRPC. However, many new androgen receptor-targeted medicines and chemotherapeutic drugs have been approved and there is currently no consensus on which patients should receive Ra-223 and when it should be administered.

We review our initial experience of using Ra-223 for mCRPC and evaluate whether pretreatment prostate specific antigen (PSA) levels correlate with completion of Ra-223 treatment. In addition, we examine change ratios of PSA, alkaline phosphatase (ALP) and bone-specific alkaline phosphatase (BAP) after the third administration to evaluate the correlation of these change ratios with completion of the subsequent Ra-223 treatment.

## Materials and methods

### Patients

This retrospective study enrolled all patients with prostate cancer who underwent Ra-223 treatment at the Japan Community Health Care Organization (JCHO) Tokyo Shinjuku Medical Center from December 2016 to May 2020. The present study was approved by the Ethics Committee of the JCHO Tokyo Shinjuku Medical Center (H30-8) and performed in accordance with relevant guidelines and regulations. We obtained written informed consent for Ra-223 treatment from all patients.

### Ra-223 treatment

All patients received Ra-223 (Bayer Yakuhin, Ltd., Japan) 55 kBq/kg body weight intravenously, every 4 weeks for up to six administrations. Ra-223 treatment was considered completed in patients who received five or six administrations. No visceral metastases were confirmed before the first administration by computed tomography (CT), given that Ra-223 is not approved in patients with visceral metastases. Blood tests were conducted after each administration. Imaging assessments including CT were performed after the third Ra-223 administration to confirm no new visceral metastases. Acute toxicity was assessed according to the Common Terminology Criteria for Adverse Events version 4.0^5^.

### Evaluation

The following background characteristics were evaluated between patient groups (complete vs. incomplete Ra-223 treatment): age, performance status (PS), treatment history, PSA, ALP, BAP levels before Ra-223 treatment and extent of bone disease. Changes in blood levels of biomarkers including PSA, ALP and BAP were compared between the two groups. Correlations between the groups were analyzed by Wilcoxon’s rank-sum tests. The Kaplan–Meier estimator and log rank test were used to assess patients’ survival fraction between the two groups. Receiver-operating characteristic (ROC) analysis was executed to analyze PSA levels before Ra-223 treatment. All analyses were performed using BellCurve for Excel (Social Survey Research Information Co., Ltd.). A *p*-value < 0.05 was considered significant.

## Results

### Patients

**Figure 1:**
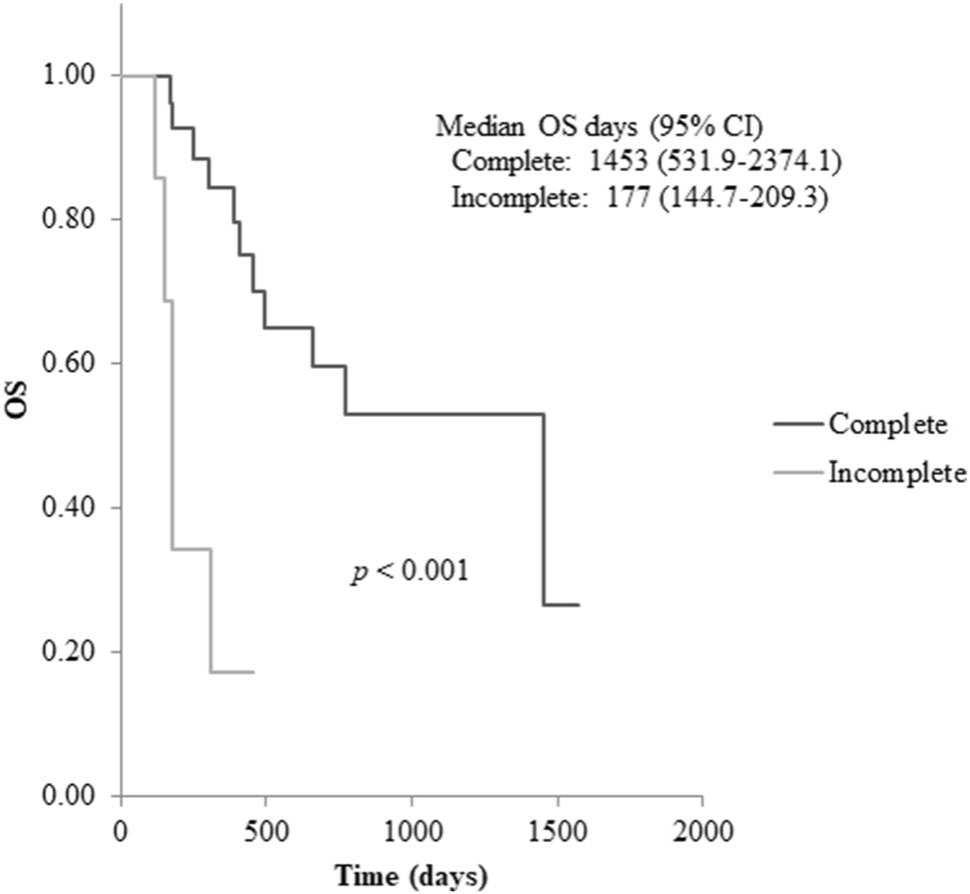
Kaplan–Meier analysis of overall survival (OS) since start of Ra-223 treatment between the complete and incomplete group. Kaplan–Meier analysis revealed significantly shorter OS for patients in the incomplete arm (*p* < 0.001). CI; confidence interval.

A total of forty patients with histologically confirmed progressive CRPC with at least two bone metastases and no known visceral metastases were enrolled in the study. The patient characteristics are shown in Table 1. Twenty-eight patients completed six administrations of Ra-223; however, visceral metastases became evident soon after the sixth administration in two patients and they might have had unknown visceral metastases during Ra-223 treatment. Two patients had five administrations, and the reason of Ra-223 discontinuation were liver metastases and bone marrow infiltration. As for the incomplete arm, two patients had four, six patients had three, one had two and one had only one administrations. The reasons of discontinuation in the incomplete arm are as follows; degrading PS in two, visceral metastases (lung, liver, spleen) in five, muscle metastases in one and lymph node metastases in two patients. Kaplan–Meier analysis revealed a significantly shortened overall survival (OS) of 1276 days in the incomplete arm (Fig. 1).

**Table 1:** Backgrounds of the study patients.

	Complete arm	Incomplete arm
**Number of activities (mean)**	5–6 (5.9)	1–4 (2.9)
**Cases**	30	10
**Age (mean)**	54–90 (70.5) years	59–86 (71.1) years
**PS**		
0	28	7
1	2	2
2	0	1
**Use of bone modifying agents**		
Yes	18	8
No	12	2
**Previous abiraterone or enzalutamide**		
Yes	21	8
No	9	2
**Previous chemotherapy (docetaxel or cabazitaxel)**		
Yes	13	8
No	17	2
**PSA before Ra-223 treatment (ng/ml) [median]**	0.01–860.25 [5.88]	22.49–2259.18 [113.15]
< 10	17	0
≥ 10, < 100	9	5
≥ 100, < 1000	2	4
≥ 1000	2	1
**ALP before Ra-223 treatment (U/L) [median]**	135–1499 [245]	136–535 [228]
**BAP before Ra-223 treatment (ng/L) [median]**	6.4–141 [12.9]	8.5–49.9 [13.1]
**Extent of bone disease**		
2–5 metastases	14	3
6–20 metastases	7	0
> 20 metastases	9	7
Superscan	0	0

PS: performance status; PSA: prostate specific antigen; ALP: alkaline phosphatase; BAP: bone-specific ALP.

The European Medicines Agency recommended contraindicating the use of Ra-223 with abiraterone acetate and prednisone/prednisolone because of an increased risk of fractures and death^6^. We therefore did not administer Ra-223 plus abiraterone after this recommendation was published, but three patients received this combination prior to publication of the recommendation.

### Safety

There was one case of grade 3 hematologic toxicity but no other severe adverse events ≥ grade 3.

### Comparison between the two groups

**Figure 2:**
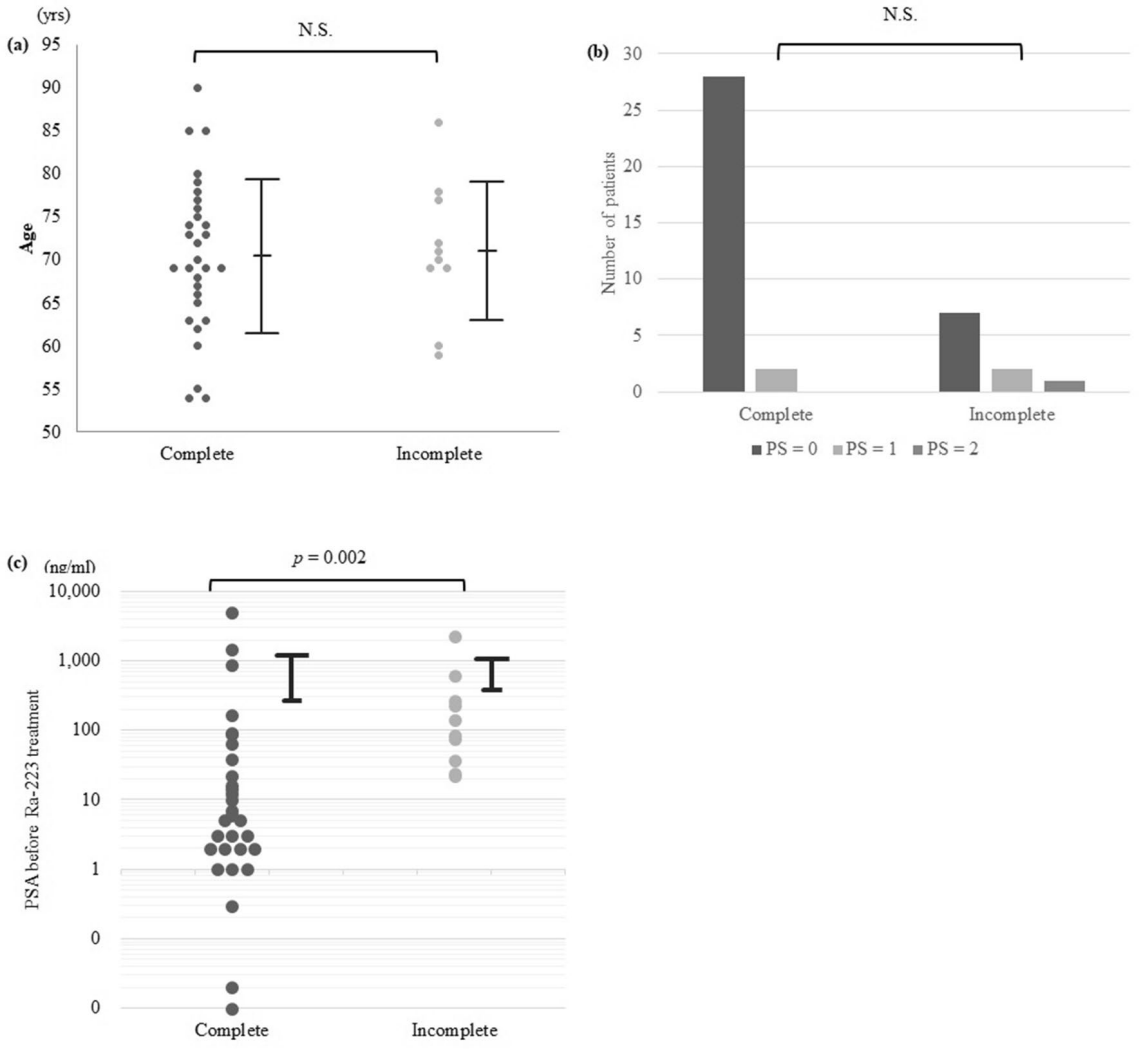
Distributions of age (**a**), PS (**b**), and PSA levels (**c**) in the complete and incomplete group (bars = standard deviations). PSA levels before Ra-223 treatment was significantly lower in the complete group compared with the incomplete group (*p* = 0.002). No significant differences were seen in age and PS between the two groups. PS; performance status, PSA; prostate specific antigen, N.S.; not significant.

The distributions of age, PS, and PSA were compared between the two groups (Fig. 2). PSA before Ra-223 treatment was significantly lower in the complete group compared with the incomplete group (*p* = 0.002). ROC analysis demonstrated that PSA cutoff value was 21.7 (Area under the ROC curve; 0.83, standard error; 0.06, 95%-confidence interval; 0.71–0.96, *p*-value; < 0.001). No significant differences were seen in age and PS between the two groups.

**Figure 3:**
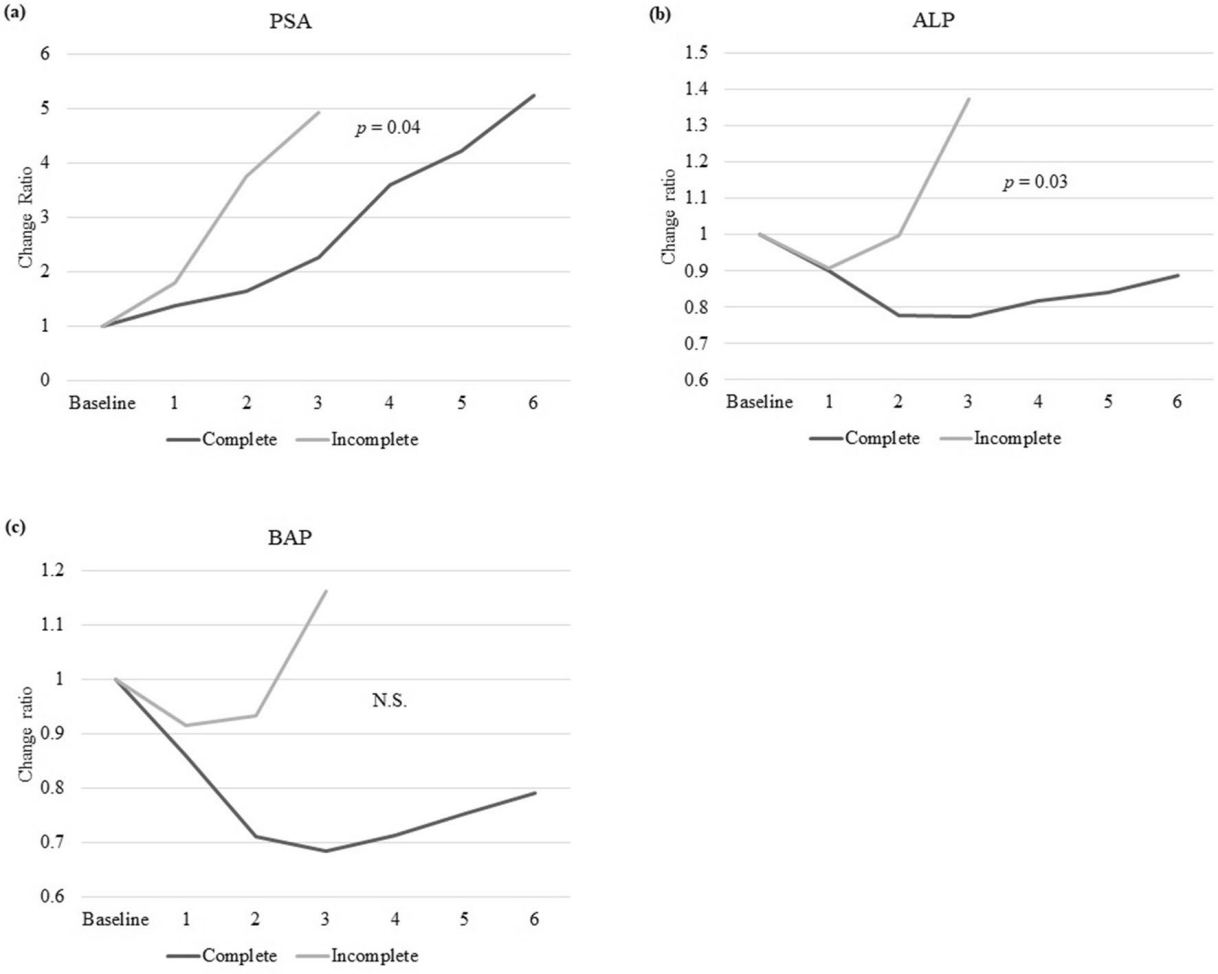
Changes in biomarkers during Ra-223 treatment. (**a**) PSA, (**b**) ALP and (**c**) BAP. *p*-values in change ratios from baseline between the complete and incomplete groups after the third administration. Mean PSA levels tended to increase throughout the entire Ra-223 treatment period in both the complete and the incomplete groups, however significant difference was seen between the two groups (*p* = 0.04). Mean ALP and BAP levels decreased after the third administration in the complete group, compared with baseline (mean change ratios 0.77 and 0.68, respectively), while levels in the incomplete group increased (mean change ratios 1.38 and 1.16, respectively). Significant difference was seen in ALP levels (*p* = 0.03), while was not seen in BAP levels (*p* = 0.08) between the two groups. PSA; prostate specific antigen, ALP; alkaline phosphatase, BAP; bone-specific alkaline phosphatase, N.S.; not significant.

Changes in the biomarkers; PSA, ALP and BAP are shown in Fig. 3. Ratios were calculated relative to the baseline before Ra-223 treatment. Mean PSA levels tended to increase throughout the entire Ra-223 treatment period in both the complete and the incomplete groups, however significant difference was seen between the two groups (*p* = 0.04). Mean ALP and BAP levels decreased after the third administration in the complete group, compared with baseline (mean change ratios 0.77 and 0.68, respectively), while levels in the incomplete group increased (mean change ratios 1.38 and 1.16, respectively). Significant difference was seen in ALP levels (*p* = 0.03), while was not seen in BAP levels (*p* = 0.08) between the two groups. There was a difference in average survival between the patients whose mean ALP levels decreased after the third administration and those did not (decrease; 892 days, increase; 688 days), however, Kaplan–Meier analysis revealed no significant difference in OS between the two groups (*p* = 0.52).

## Discussion

Ra-223 is the first alpha emitter with a clinical role in the treatment of cancer, and the first radiopharmaceutical shown to both improve OS and increase the time to the first symptomatic skeletal event^7^. Docetaxel was approved for the treatment of CRPC in 2008, followed by enzalutamide, abiraterone and cabazitaxel in 2014; however, the choice and timing of therapy for CRPC remains controversial. Ra-223 treatment was started in our hospital in 2016. At that time, most patients had advanced prostate cancer before starting Ra-223 therapy, and had therefore already received all viable treatment options, including chemotherapy, which had been judged to be ineffective. Although we wanted to administer Ra-223 treatment to these patients sooner, the system was not yet established. These patients also had so many bone metastases. We subsequently enrolled patients with relatively stable disease, fewer bone metastases, and a PSA level < 100 ng/mL, or those who still had other treatment options available, once the Ra-223 system had been established and patients could therefore be treated sooner. The pivotal phase III ALSYMPCA trial included patients with pain due to bone metastases^7^. McKay et al. reported that survival tended to be longer in patients with little or no pain compared with those with moderate or severe pain^8^. Furthermore, all measures of survival were longer in patients with PS 0 compared with patients with PS 1 or 2^8^. More frequent Ra-223 treatment has also been associated with a better prognosis^8^.

Regarding safety, Ra-223 treatment was well tolerated, and severe adverse events are unlikely to occur if the appropriate criteria for radiopharmaceutical administration are met. In the current study, only one patient experienced grade 3 hematologic toxicity. This patient had progressive prostate cancer, but his PS was good and he had no visceral metastases. We assumed that he might have developed anemia irrespective of the receipt of Ra-223 treatment.

As for patient backgrounds, pretreatment PSA levels were significantly lower in patients in the complete group, but there were no differences in age and PS between the two groups. Nakatani et al. also reported a difference in PSA levels before Ra-223 treatment between the completed and uncompleted arms^9^. Low PSA levels reflect weaker disease, suggesting that Ra-223 treatment should be considered in patients with early-stage prostate cancer. However, inability to complete all treatment cycles does not mean the patient fails to experience any benefit. Thus, patients should not be denied access to Ra-223 treatment as based on high PSA levels. Previous studies have shown that imaging based prognostic biomarkers might help to identify patients with favorable survival after Ra-223 treatment. Dittmann et al. reported that quantitative single-photon emission computed tomography (SPECT)/CT of bone scans performed at baseline is prognostic for survival^10^. Fosbøl et al. demonstrated that the Bone Scan Index (BSI) is a promising biomarker for prognostication of OS^11^. These are useful, however, SPECT/CT cannot easily be performed in our institution and not all bone scintigraphies are equipped with the additional BSI software. Compared with imaging based biomarkers, blood test data is easy to use.

Although baseline factors of the completion are very important, in clinical practice, it is also essential to judge whether the Ra-223 treatment should be continued or quitted and changed to other treatment. As widely known, increasing PSA during Ra-223 treatment cannot be used as a clue to evaluate that the on-going Ra-223 treatment is ineffective, and so, we compared changes in blood levels of biomarker between the two groups. In terms of biomarkers, PSA levels tended to increase during Ra-223 treatment. PSA levels had only decreased from baseline in nine of the thirty patients in the complete arm after the final administration in the current study, while Nakatani et al. reported decreased PSA levels in two of five patients^9^. These were in accordance with the results of the pivotal phase III ALSYMPCA trial^7^. The mean change ratios in PSA after the third administration was significantly lower in the complete compared with the incomplete group. ALP and BAP show similar trends of decrease and gradual increase. BAP is an isoform of ALP and a relatively specific marker for osteogenesis^12^, thus explaining the similar trends. However, the degrees of decrease and increase of both ALP and BAP differed between the two groups of the current study. After the third administration of Ra-223, ALP and BAP levels had decreased to below baseline levels in the complete group, while levels in the incomplete group had increased to above baseline, although the significant difference was seen only in ALP (*p* = 0.03). Nakatani et al. reported large changes in LDH and ALP after starting Ra-223 treatment^9^. This study showed no significant difference in OS between the patients whose mean ALP levels decreased after the third administration and those did not, while Sartor et al. have shown that patients with ALP decline from baseline to week 12 had 55% lower risk of death versus those with no confirmed ALP decline^13^. This difference may be due to the difference in the number of patients; the number of patients we studied was too small. The main reason for discontinuing Ra-223 treatment in patients in the incomplete arm was visceral metastases revealed by imaging assessment. ALP and BAP are markers of the progression of bone metastases, but not visceral metastases. The significant increases of PSA and rapid increases of ALP and BAP in the incomplete arm might indicate that Ra-223 has a weak effect on bone metastases in patients with visceral metastases.

This study was limited by its small sample size and short follow-up period. Future studies with larger sample sizes and longer follow-up periods are therefore needed to validate the findings.

In conclusion, we report on our initial experience of using Ra-223 for mCRPC. The results suggest that Ra-223 treatment should be considered in patients with early-stage prostate cancer with low PSA levels. Rapid increases in ALP and BAP in patients in the incomplete arm with visceral metastases suggest that Ra-223 treatment might have a weak effect on bone metastases in patients with visceral metastases. Changes in PSA and ALP during Ra-223 treatment might be a useful indicator of the likelihood of completing Ra-223 treatment, and of the subsequent patient prognosis.

## Data Availability

The datasets used and analyzed during the current study are available from the corresponding author on reasonable request.
