# Author Correction: Pretreatment PSA levels affects the completion rate of Ra-223 treatment

**DOI:** 10.1038/s41598-021-96170-5

**Published:** 2021-08-16

**Authors:** Nobuko Utsumi, Hiromasa Kurosaki, Kosei Miura, Hiroki Kitoh, Koichiro Akakura

**Affiliations:** 1 Department of Radiation Therapy, Japan Community Healthcare Organization Tokyo Shinjuku Medical Center, 5‑1, Tsukudocho,, Shinjuku‑Ku, Tokyo, 162‑8543 Japan; 2 Department of Urology, Japan Community Healthcare Organization Tokyo Shinjuku Medical Center, 5‑1, Tsukudocho, Shinjuku‑Ku, Tokyo, 162‑8543 Japan

Correction to: *Scientific reports* 10.1038/s41598-021-86033-4, published online 19 March 2021

The original version of this Article contained errors in Figure 1, where the data in the inset key description for “Complete” and “Incomplete” was incorrectly swapped.

The original Figure 1 and accompanying legend appear below.

**Figure 1:**
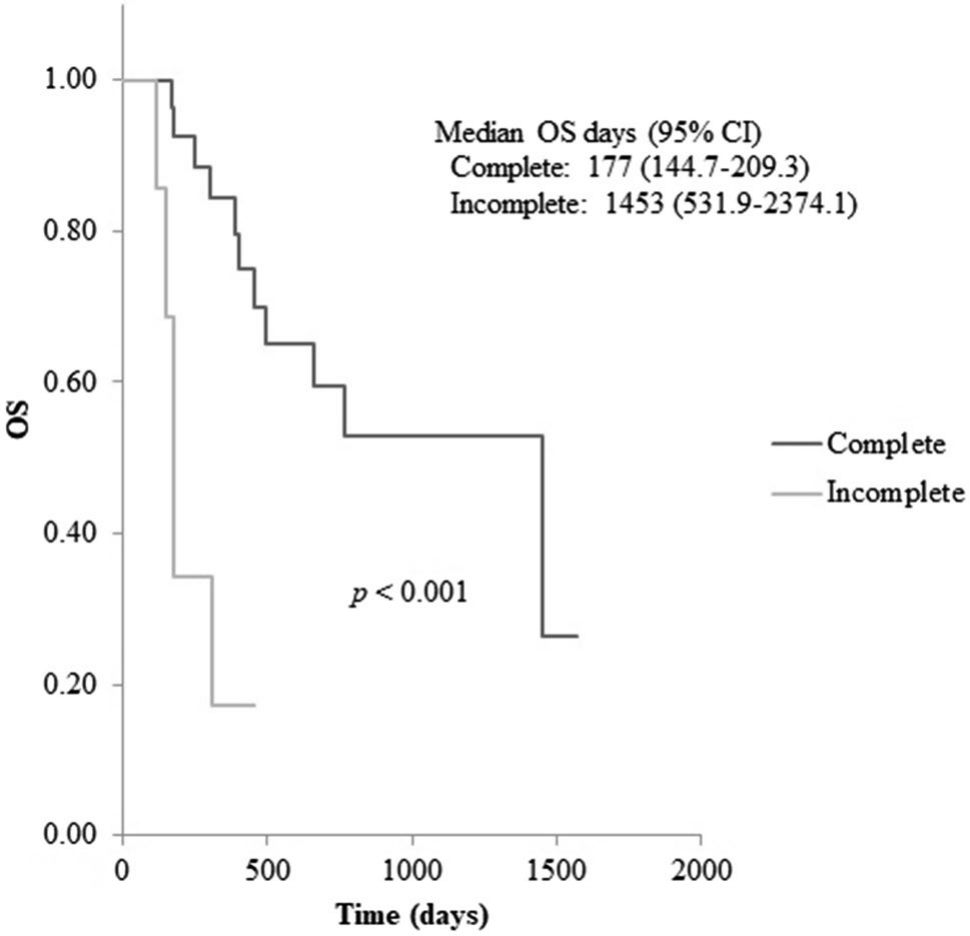
Kaplan–Meier analysis of overall survival (OS) since start of Ra-223 treatment between the complete and incomplete group. Kaplan–Meier analysis revealed significantly shorter OS for patients in the incomplete arm (*p* < 0.001). CI; confidence interval.

The original Article has been corrected.

